# ECO-SEOM-SEEO safety recommendations guideline for cancer patients receiving intravenous therapy

**DOI:** 10.1007/s12094-020-02347-1

**Published:** 2020-04-30

**Authors:** I. Magallón-Pedrera, J. Pérez-Altozano, J. A. Virizuela Echaburu, C. Beato-Zambrano, P. Borrega-García, J. C. de la Torre-Montero

**Affiliations:** 1 Oncology Nursing Service, Vall d’Hebron University Hospital, Spanish Society of Oncology Nursing (SEEO), Barcelona, Spain; 2 Medical Oncology Service, Virgen de los Lirios de Alcoy Hospital, Spanish Society of Medical Oncology (SEOM), Alicante, Spain; 3 Medical Oncology Service, Virgen Macarena University Hospital, Foundation for Excellence and Quality in Oncology (ECO), Seville, Spain; 4 Medical Oncology Service, Virgen Macarena University Hospital, Spanish Society of Medical Oncology (SEOM), Seville, Spain; 5 Medical Oncology Service, Hospital San Pedro Alcántara, Foundation for Excellence and Quality in Oncology (ECO), Cáceres, Spain; 6 Comillas Pontifical University, San Juan de Dios School of Nursing and Physical Therapy, Spanish Society of Oncology Nursing (SEEO), Alberto Aguilera, 25, 28015, Madrid, Spain

**Keywords:** Cancer therapy, Safety, Venous access, Decision algorithm, Evidence-based recommendations, Clinical practice survey

## Abstract

**Purpose:**

Cancer patients require implantation of venous access devices to meet their personalized therapeutic needs, which are often complex due to the nature of the medication and the disease status. Therefore, it is essential to have standardized protocols that guarantee the best results in health and patient safety.

**Methods:**

To learn about the availability of protocols and aspects related to safety in clinical practice and to detect possible opportunities for improvement, a survey has been conducted in various Spanish hospitals, in addition to a review of the evidence regarding the various devices available and complications associated with the administration of chemotherapy.

**Results:**

As a result of both analyses, the Foundation for Excellence and Quality in Oncology (ECO), the Spanish Society of Medical Oncology (SEOM), and the Spanish Society of Oncology Nursing (SEEO) have developed a catheter selection algorithm based on patient characteristics and treatment to facilitate the clinical decision-making process, as well as some recommendations aimed at ensuring patient safety and rational use of available resources.

**Conclusions:**

In conclusion, both the venous access catheter selection algorithm and the proposed recommendations aim to respond to the needs revealed in clinical practice and to become an integrable tool in electronic prescription systems to offer homogeneous criteria for action in cancer patients that require venous access, optimizing the use of available health resources with the highest safety and quality of life for the patient.

## Introduction

Owing to its chronic nature and significant impact on health, cancer generates certain complex needs in patients that require multidisciplinary and personalized care through strategies that integrate the patient's perspective, under the coordination of the Medical Oncology Department and in collaboration with the other healthcare professionals involved in its care circuit [1].

One of the most disturbing aspects for these patients is the need to perform multiple painful venous punctures for analytical extractions and administration of cytotoxic agents, antibiotics, blood derivatives or nutritional supplements [2]. To reduce the negative impact of such punctures, it is beneficial to have a stable venous access that can be reused, facilitating both the administration of drugs and appropriate monitoring of the patient's condition and reducing the anxiety associated with this procedure [3].

To achieve this, there are many devices, for both central and peripheral venous access [2–4], it being a prerequisite for all of them that they should be reliable and safe to use, since there are intrinsic complications of both the medication and the procedure that must be adequately addressed to achieve the best clinical results. It is therefore essential to analyse the different vascular access options available and to establish appropriate criteria for selecting the most suitable device in each case, taking into account key aspects such as the physicochemical characteristics of the therapy and its duration, the physical condition and history of the patient, the resources and devices available or the integrity of the patient's vascular system and their personal preferences [5]. It is also important to take into account the experience and level of training of the professionals in charge of their insertion and care, as it has been established that the greater the specific professional preparation, the fewer associated problems [6].

## Objective

In this context, the Foundation for Excellence and Quality in Oncology (ECO), the Spanish Society of Medical Oncology (SEOM), and the Spanish Society of Oncology Nursing (SEEO), as part of their commitment to cancer patients, have carried out the project “IniciatiIVas. Oncology patient safety in intravenous therapy: situation and recommendations” to respond to the needs raised through the drafting of recommendations based on both evidence and analysis of the current situation of Spanish hospitals to optimize the use of vascular access catheters in cancer patients, with special attention to the identification of critical points to increase opportunities for improvement in terms of the prevention and management of possible adverse effects, and to increase patients' quality of life.

## Methods

### Clinical practice survey

To find out the current situation regarding the management of cancer patients requiring intravenous therapy, a nationwide population survey was prepared online consisting of a total of 14 questions, some of them multiple-choice, aimed at analysing and quantifying the incidence of adverse effects related to the route of administration, comparing existing protocols and exploring available care resources. The survey was prepared by an expert committee composed of six members from the above-mentioned scientific societies and subsequently disseminated for completion by all the practitioners concerned. The spectrum of professionals consulted included three main categories, which were Medical Oncology Department heads, Nursing supervisors of that unit and a third group consisting of Medical Oncology Section heads and staff belonging to both the Nursing Department of the Day Hospital and the Clinical Trials Unit.

The estimation of the statistical representativeness of the sample at both the national and autonomous community levels was determined with a 95% confidence interval, an accuracy of ± 5% and a percentage of necessary replacements of 10% as the assumption of maximum uncertainty in each of the responses obtained.

Statistical data processing was performed using the IBM SPSS software package (IBM Corp. Released 2011. IBM SPSS Statistics for Windows, Version 20.0. Armonk, NY: IBM Corp.) showing results as absolute frequencies (number of cases) and percentages (%) for categorical variables and as mean ± standard deviation (SD) for continuous variables.

### Evidence review

In addition, an updated bibliographical review was carried out on the main aspects relating to intravenous therapy and venous access devices used in oncology, as well as the protocols currently in force in this field, using the main databases available in the health field and other reference documents endorsed by health agencies and bodies of recognized national and international scientific value. The thematic blocks that made up this review were also raised by the members of the scientific committee, including aspects relating to the physiological implications of vascular access, the choice of venous capital for vascular access in oncology, the types of catheters available based on the duration of therapy, the procedures necessary for the cannulation of each of these catheters and the most relevant safety issues regarding the use of these devices in the cancer patient.

## Results

### Clinical practice survey

#### Profile of participants

The survey was answered by a total of 178 health professionals belonging to 98 Spanish public, private or state-subsidized hospitals, with the results being statistically representative at a national level and, additionally, at a regional level in the case of seven autonomous communities.

A detailed description of the sociodemographic characteristics of the participants is shown in Table 1.

**Table 1:** Main sociodemographic characteristics of the participants in the multicenter population survey

Years of experience (mean, SD)	22.3 (7.1)
Age (mean, SD)	49.76 (9.29)
Sex (%, *n*)	
Male	36.0 (64)
Female	**64.0 (114)**
Position (%, *n*)	
Head of department	**43.8 (78)**
Nursing Supervisor	29.2 (52)
Other*	27.0 (48)
Work center (%, *n*)	
Private hospital	7.3 (13)
Public hospital	**83.4 (150)**
State-subsidized hospital	8.4 (15)
Number of beds (%, *n*)	
Under 200 beds	14.6 (26)
200–500 beds	**39.3 (70)**
501–1000 beds	30.9 (55)
Over 1000 beds	15.2 (27)
Autonomous community (%, *n*)**	
Andalusia	5.6 (10)
Aragon	3.4 (6)
Balearic Islands	2.8 (5)
Canary Islands	5.6 (10)
Castile and Leon	6.2 (11)
Castile La Mancha	3.9 (7)
Catalonia	14.0 (25)
Ceuta and Melilla	0.6 (1)
Community of Madrid	**18.0 (32)**
Chartered Community of Navarre	3.4 (6)
Valencian Community	**18.0 (32)**
Extremadura	2.2 (4)
Galicia	5.1 (9)
La Rioja	1.1 (2)
Basque Country	4.5 (8)
Principality of Asturias	1.7 (3)
Region of Murcia	3.9 (7)

Bold numbers are the maximum (peak) numbers at the final analysis

*This category included Medical Oncology Section heads and staff from both the Day Hospital Nursing Department and the Clinical Trials Unit

**No response was obtained in hospitals corresponding to Cantabria

#### Overall results

With regard to issues relating to the safety of cancer therapy, the survey results revealed that virtually all participants (99.4%) considered the prevention of associated adverse effects, such as extravasations, phlebitis and bacteremia, to be a priority in appropriate patient management. Regarding the role of the various clinical areas involved in their prevention, the majority of respondents attached high importance to the Nursing (85.4%) and Medical Oncology (48.9%) Departments, with the role of the Preventive Medicine Department being less relevant (29.2%). However, while more than half of the participants (63.5%) confirmed the availability of intravenous therapy equipment for cancer patients at their hospital, a significant percentage (60.1%) reported not having a record of adverse events associated with this type of therapy. For those who did confirm having this record, the majority of participants indicated that these safety data are collected mainly through the oncohematology day hospital (92.9%) and, to a lesser extent, by the hospitalization units (39.4%), quantifying the incidence of extravasations and bacteremia at an average of 7 events each year, being higher in the case of phlebitis with an average of 23 events each year. Additionally, the results of the survey revealed that although intravenous therapy equipment for cancer patients may not be available, in half of the cases (52.2%) there is intravenous therapy equipment for patients of other characteristics.

With regard to aspects relating to the application of algorithms and protocols concerning the use of central venous access devices for the prevention of adverse events in cancer patients, approximately half of participants (58.4%) stated having at their disposal established guidelines for action in which the duration of treatment (94.2%) and the vesicant nature of the medication (93.2%) are considered among the selection criteria for the catheter. Regarding the validation and approval of these protocols, the majority of respondents who confirmed their availability stated that this is carried out in most cases by the Medical Oncology Department (81.7%), although a significant percentage of participants considered that the Oncology Nursing Department is also actively involved (69.2%). Regarding the prescription of the infusion system, again the majority of respondents agreed that it is carried out interchangeably by both departments (48.1%). For those participants who reported not having specific protocols and algorithms, most agreed that this absence relates to validation issues in the unit or hospital (64.9%), followed by other factors such as lack of training by Nursing staff for cannulating peripherally inserted central catheters (PICCs) (50.0%), insufficient information and awareness (41.9%) or even the lack of guidelines endorsed by scientific societies (29.7%). In this regard, almost all respondents (98.9%) considered it necessary to have a nationally applicable cancer patient infusion therapy algorithm validated by the relevant scientific societies.

Regarding the different devices available for venous access in oncology, the survey results revealed that the parties responsible for cannulating each of them vary substantially. Thus, the majority of respondents (87.2%) said that the Nursing Department is responsible for implanting the peripheral catheter, while for the placement of PICCs, the parties responsible are mostly (80.0%) the Anesthesia or Vascular Surgery Departments or Venous Access Unit, followed by the Nursing Department (65.8%). In the case of reservoirs, the majority (87.7%) also agreed that the Anesthesia, Vascular Surgery and Oncology Departments, Intensive Care Unit or Venous Access Unit are responsible for their placement.

Regarding training for the implementation of PICCs by the Nursing Department, 39.4% reported that despite having a protocol, staff do not have the necessary training for their insertion. However, most participants (96.1%) said that they would be interested in implementing an algorithm validated by scientific societies that would allow the Nursing Department to prescribe the infusion system, in addition to providing them with the appropriate training to carry out the placement of PICCs.

In summary, the survey results reflect the following deficiencies in Medical Oncology Units regarding the safety of intravenous therapy:

- Absence of record of adverse events associated with intravenous cancer therapy.
- Absence of specific equipment for administering intravenous cancer therapy.
- Absence of a venous access selection protocol/algorithm, it being considered of great importance to have one at a national level approved by scientific societies.
- Lack of information among healthcare professionals on the professional competence of nursing staff for the insertion of PICCs and lack of specific training of such staff.

### Evidence review

#### Venous access catheters for use in cancer patients

There are numerous types of vascular catheters with different characteristics depending on the method of insertion, indication, material, gauge, length, location, tip termination, number of lumens they contain or associated risk of complications. Generally speaking, according to their location, catheters can be classified as peripheral or central, with the choice of catheter being determined by various factors such as the duration of implantation, the pharmacological nature of the infusion, the specific characteristics of the patient or the assessment of the possible risks associated with their use [7].

#### Peripheral venous access catheters

There are currently two main types of peripheral access venous catheters, short catheters [8] and the medial venous midline catheters (MVCs), which enable the duration of infusion therapy to be extended [4]. The former are devices 3 to 6 cm long, usually inserted into the veins of the forearm, indicated as short-term venous accesses when the therapy is expected to last less than 6 days [8]. The latter are devices ranging from 8 to 25 cm, usually placed in the brachial or cephalic vein of the arm, indicated for therapies lasting at least 6 weeks [4].

Both can be cannulated by either doctors or nursing staff, the necessary equipment being inexpensive and the insertion technique relatively simple, which makes them a widely used option in cancer patients. However, existing recommendations discourage their use for the administration of vesicant and hyperosmolar agents and in prolonged infusions (> 60 min) [9] as infiltration into adjacent tissues may result in tissue necrosis. However, it should be noted that MVCs allow the administration of fluids with a low irritant capacity for up to 7 days and have been associated with lower rates of phlebitis than short catheters [4].

According to the available studies, complications associated with the use of peripheral catheters occur in around 35–50% of cases before the end of the expected time of use, so it is recommended to replace them after 72–96 h [5]. However, in patients who remain hospitalized there is increasing evidence in favor of withdrawing them by clinical indication, i.e. after the end of treatment or in the event of complications, since routine replacement entails increased economic costs, care burden and discomfort caused to the patient [10], with the possibility of changing the catheter at each visit being considered for outpatients.

#### Central venous access catheters

Central venous catheters (CVCs) are used for various purposes, such as infusion of medicines and blood derivatives, hemodialysis, blood sampling, and hemodynamic monitoring, and can remain implanted for weeks and even years. The choice of central catheter type for each situation should be based on criteria such as duration of treatment, patient characteristics, infusion type and device characteristics, with duration of implantation being one of the most important factors [5]. To this end, although there is no clear definition of what should be considered short- or long-term, a recent study sets the limit at 30 days [7], which allows recommendations to be made to use PICCs for durations of implantation shorter than this period and tunneled catheters or PICCs for longer durations of implantation [11].

PICCs are central venous catheters usually inserted percutaneously through the basilic, cephalic, or brachial vein of the upper limb with their distal end at the junction of the superior vena cava and the right atrium. They are considered an effective alternative to traditional central catheters for many indications, both in the short term when the catheter must remain implanted for more than 6 days or peripheral access is not possible and in the long term when the duration of treatment is 6 months to 1 year and it is not possible to implant a reservoir [12], owing to their safety, ease of insertion and reduced number of complications [7].

One of the main advantages they offer compared with other central catheters is that they can be inserted by Nursing staff at the bedside, the use of ultrasound being recommended, which significantly reduces complications [13], as well as reducing the number of failed punctures, being faster and more comfortable for the patient, [7] and the costs associated with the procedure [13–15]. Specifically, a study conducted in Spain showed that ultrasound-guided cannulation of PICCs by nursing staff in oncology and hematology patients is associated with a high insertion success rate (89.7%), with the catheter remaining implanted for on average 92 days and very low rates of complications [16]. Central inserted central catheters (CICCs) are catheters that are inserted from a central vein such as the subclavian, jugular, or femoral vein and whose distal end is located in the superior or inferior vena cava, near the junction with the right atrium. Generally speaking, they are divided into tunneled or non-tunneled catheters, the choice of which in each case will be determined mainly by the length of time they must remain implanted. Specifically, non-tunneled CICCs are indicated for short durations of implantation not exceeding 4–6 weeks, being implanted percutaneously at the bedside by the relevant professional, while tunneled CICCs are implanted surgically via a subcutaneous route, and may be implanted for longer periods (up to 12 months) [7].

Regarding their insertion, the use of two-dimensional ultrasound has been shown to reduce mechanical complications and the number of attempts required for successful cannulation compared with standard placement with reference points, so it is recommended to use this technique for cannulation [17].

Implanted venous access devices consist of a reservoir from which a central catheter departs and flows into a central vein near the heart. Their insertion is performed subcutaneously, usually in the chest or upper part of the arm, by surgery^11^ using a needle specifically designed not to perforate it called a Huber needle, and it is recommended that it be of the smallest possible gauge according to the prescribed therapy [18]. However, although they are associated with a low infection rate and reduce the limitations on patient mobility owing to the absence of an external component [19], their cost is high and their insertion requires more resources and time than other central venous access options.

#### Safety aspects relating to the use of venous capital in oncology

In the cancer patient population, the risk of catheter-related complications is potentially increased, owing to the presence of immunosuppression, thrombocytopenia and coagulopathy from both the disease and its treatment, increasing the incidence of infections and thrombosis [20]. On the other hand, most of the time the treatments used are potentially harmful to the tissues, with the consequent risk of extravasation and complications [21].

##### Main complications associated with the medication

Extravasation is a potential accidental complication associated with the administration of chemotherapy with serious consequences for the patient, as it may result in tissue necrosis, associated with various factors such as the characteristics of the chemotherapy agent (e.g. vesicant potential, volume and concentration administered, rate and duration of infusion) or the patient (e.g. access to small or fragile veins, presence of lymphoedema or obesity or history of multiple venous punctures) [21].

Its prevalence varies between around 0.1–6% when administered through a peripheral catheter and between 0.26–4.7% if a central catheter is used [21], so the administration of irritant or vesicant antineoplastics by peripheral infusion pumps is not recommended, unless reduced-pressure pumps are used [22], and comprehensive monitoring is recommended if central access is used, given the potential risk of accumulation in the mediastinum, pleura or subcutaneous area of the chest [23].

##### Main catheter-associated complications

Infections are one of the most serious complications to consider among cancer patients, owing to both the treatment and malignancy conditions of the disease [24] and the conditions related to the venous access itself [20]. Regarding their incidence according to the type of central access used, the evidence is still controversial, as some studies performed in cancer patients showed significantly lower rates with PICCs versus CICCs (1.23 vs. 5.3/100 days of catheter use) [25] or a lower incidence with PICCs in outpatients [26], while other data suggest that in the short term the incidence of infection is similar.

On the other hand, cancer patients often require the simultaneous administration of incompatible infusions, requiring the use of multi-luminal catheters with a larger diameter, and therefore also a higher risk of thrombosis [27]. Comparing their incidence on the basis of the central access used, PICCs appear to be associated with a higher risk of deep vein thrombosis than CICCs (OR 2.55 [1.54–4.23]; *p* < 0.0001); however, it should be noted that the latter present a higher risk of central vein thrombosis [20]. Additionally, the thrombosis rate has been shown to decrease significantly when a mono-luminal PICC is chosen or smaller-diameter catheters are used, and it is recommended that the catheter occupancy with respect to the vein should not exceed 45% [28].

Likewise, occlusion of the catheter lumen is a fairly common complication associated with venous access devices, occurring in 14–36% of cases of prolonged use and around 10% of cases of short-term use. The causes of a lack of reflux may be purely mechanical or related to the appearance of drug precipitates or fibrin sheaths around the tip of the catheter, the latter being one of the most common causes, and may develop even within the first 24 h after implantation of the device [29].

##### Clinical and economic impact of complications in oncology and importance of prevention

According to the information provided by ECO, SEOM and SEEO, there are approximately 150 centers in Spain that administer oncology therapy intravenously. Taking into account the incidence revealed by the survey for each of the main related complications, and extrapolating the costs involved in the management of these events reported in US hospitals, the approximate annual costs amount to €17,221,000 [30] for the management of bacteremia resulting from the catheter, €1,257,400 for the resolution of phlebitis [31] and €15,635,000 for the management of moderate extravasations, multiplying almost tenfold in the case of severe extravasations [32], which undoubtedly impose a huge burden on our health system.

There is evidence that the application of preventive measures in a protocolized manner by a well-trained team reduces by up to 7 times the incidence of complications [33], such as those adopted in a multicenter study in Michigan based on prior hand hygiene, the use of maximum barrier measures, skin disinfection with chlorhexidine, avoiding venous access through the femoral vein, and removing unnecessary routes, which showed a 66% reduction in the rate of infections [7]. Based on this multifactorial strategy, in our country the “Bacteremia Zero" project has revealed that, in the two years following its implantation, the incidence of CVC-related bacteremia in Intensive Care Units (ICUs) fell by 50% in 68% of the units [34], which represents a potential benefit that could be extrapolated to cancer patients with these devices implanted. In addition, the use of antibiotic-impregnated catheters has been shown to be a cost-effective strategy in reducing the rate of catheter-associated bacteremia [17], with the use of PICCs with antibiotic coatings being an additional preventive measure in high-risk situations such as cancer patients [24].

On the other hand, regarding the insertion of the catheter it is recommended that it be ultrasound-guided against rather than using the blind technique, which substantially reduces the number of attempts, verifying the location of the catheter tip by chest X-ray or intracavitary electrocardiographic method [7].It is also recommended that soft, flexible catheters made of radiopaque material for radiological monitoring which do not cause venous thrombosis or release harmful substances when in contact with the treatment should be used [35].

In addition, the regular washing of the catheter, both after use and maintenance if not used for a long time, is paramount in the prevention of possible obstructions, with the use of 3 ml solutions of 0.9% NaCl or 60 IU/3 ml unfractionated heparin sodium being recommended [36].

##### Nursing competence in PICC insertion

With regard to the placement of PICCs and other vascular accesses, there is no doubt that the constant technological advances that have taken place in the health sector, such as the development of the ultrasound-guided insertion technique, are an important catalyst in increasing the number of interventions and capacities of Nursing Departments, opening a new area of training for the specialty.

With regard to training, supervision and skills acquisition, the current literature available on CVCs does not establish a fully standardized program for professionals in training. Some authors consider nursing competence in PICC insertion to be adequate when staff have a minimum of 2 years’ experience, while others consider it to be after 25 or more insertions of this type of catheter [37]. However, what has been shown is that a systematic training process, including prior training in ultrasound, reduces mechanical and infectious complications in the patient [38]. Therefore, standardized education, simulation practice and supervised insertions are key tools to ensure safe and competent practice in the insertion of PICCs, and there is no doubt that the role of Nursing staff is increasingly active in this regard, with the positive clinical and economic impact that this entails.

## Discussion and conclusions

The results of the clinical practice survey conducted in almost a hundred Spanish hospitals reveal that issues relating to the safety of cancer therapy, such as the prevention of extravasations, phlebitis, and thrombosis, are a priority for healthcare professionals in the proper management of the patient, with the role of the Oncology Nursing and Medical Oncology Department being particularly relevant.

However, these same results reveal important unmet needs, such as the adequate recording and control of safety events relating to the administration of antineoplastics, the availability of protocols and approved decision algorithms for the administration of these therapies and their correct application in clinical practice, or the adequate training of Oncology Nursing staff in the placement of venous access devices, especially in the case of PICCs, to optimize their use with the consequent benefits both for the incidence of complications and the costs associated with them. Thus, although most participants indicated that there are indeed records of adverse events made by the staff of the Oncohematology Day Hospital, the percentage is much lower in the Hospitalization Units, indicating an important need to improve the monitoring of these events in the departments where they mainly occur and optimize their management. On the other hand, a high percentage of hospitals indicated that they do not have protocols for managing infusion systems in cancer patients, either because there is no awareness of their importance or because the guidelines and selection criteria are not supported by scientific societies at a national level. In this regard, it is also worth reflecting that, presumably, even though the centers have action protocols, these are not always complied with, since the reported rates of adverse events are still high. Also, although there is increasingly more training in the insertion of PICCs and Midlines by Oncology Nursing, it should be noted that this constitutes an advanced skill that is not covered in the basic studies of the specialty, so it is usually taken on by other hospital services such as anesthesia or Vascular Surgery Departments or the Venous Access Unit, or even replaced by the use of peripheral catheters incorrectly with the consequent complications. However, taking into account its proven advantages over the incidence of adverse events compared with other central venous access devices with the consequent reduction of costs associated with the procedure and its complications [7], it is essential to integrate its use into a validated algorithm that allows the Nursing Department to carry out the prescription of the infusion system.

Regarding the choice of catheter, as previously highlighted, there is no optimal vascular access device for all patients, but the most appropriate device will depend on the specific therapeutic needs and risk factors of the patient, as well as the therapy to be administered [8]. In this context, there are numerous venous access catheter selection algorithms published by various prestigious societies [39–42]. However, despite all of them having selection criteria based on the best published scientific evidence, including the use of antineoplastic drugs, none of them are specifically applicable to cancer patients.

**Fig. 1:**
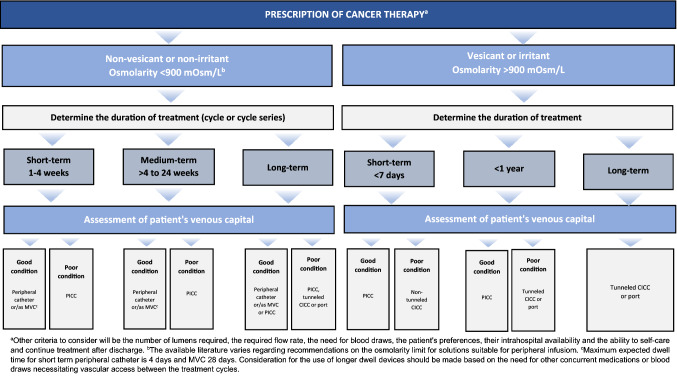
ECO-SEOM-SEEO algorithm for venous access catheter selection in cancer patients
- A series of agreed recommendations aimed at ensuring both patient safety and the rational use of available resources in intravenous oncology therapy and practical suggestions related to the catheter insertion process, the prevention of complications during the process, and adequate long-term maintenance and actions should unwanted events occur (Fig. 2).

In view of the above-mentioned needs and the evidence currently available, the scientific societies involved in this initiative have developed:

- A proposal for a catheter selection algorithm based on the characteristics of the infusion, the required duration of treatment and the clinical status of the patient (Fig. 1), to which is attached the classification proposed by the VIA scale to assess the venous capital of the patient (Table 2), a table with the advantages and disadvantages of the main CVCs used in cancer patients (Table 3) and a classification of the main vesicant, irritant and possibly irritant antineoplastics to facilitate the clinical decision-making process (Table 4).

**Fig. 2:**
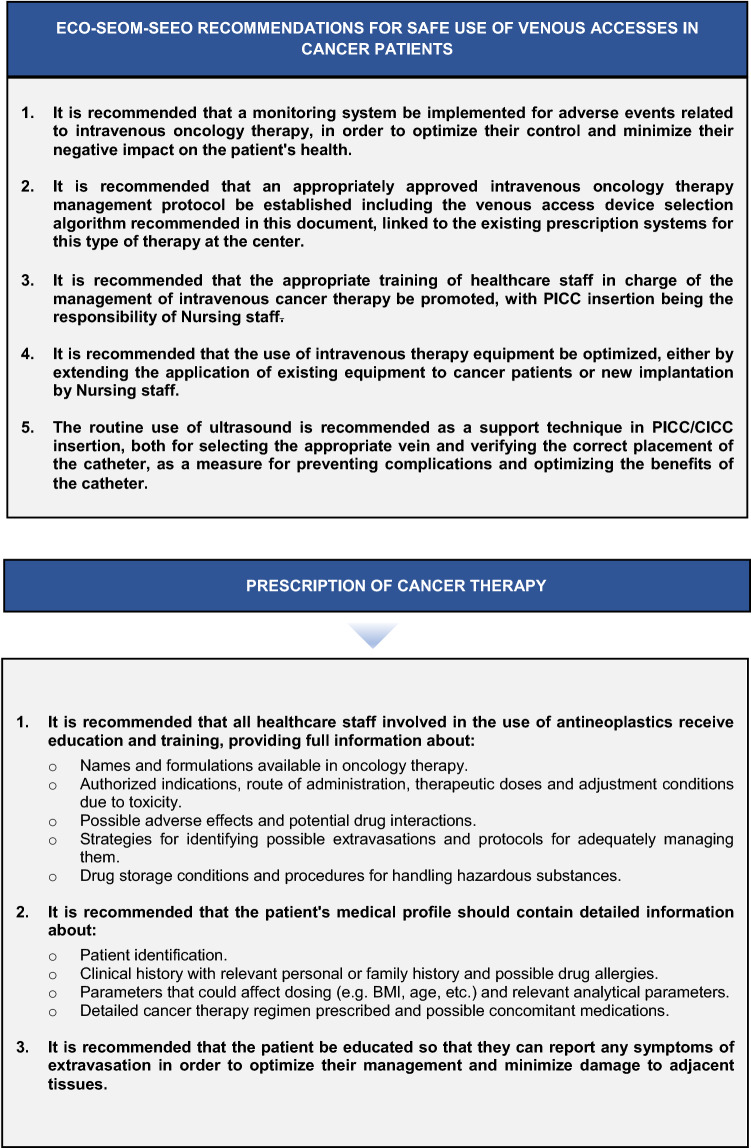

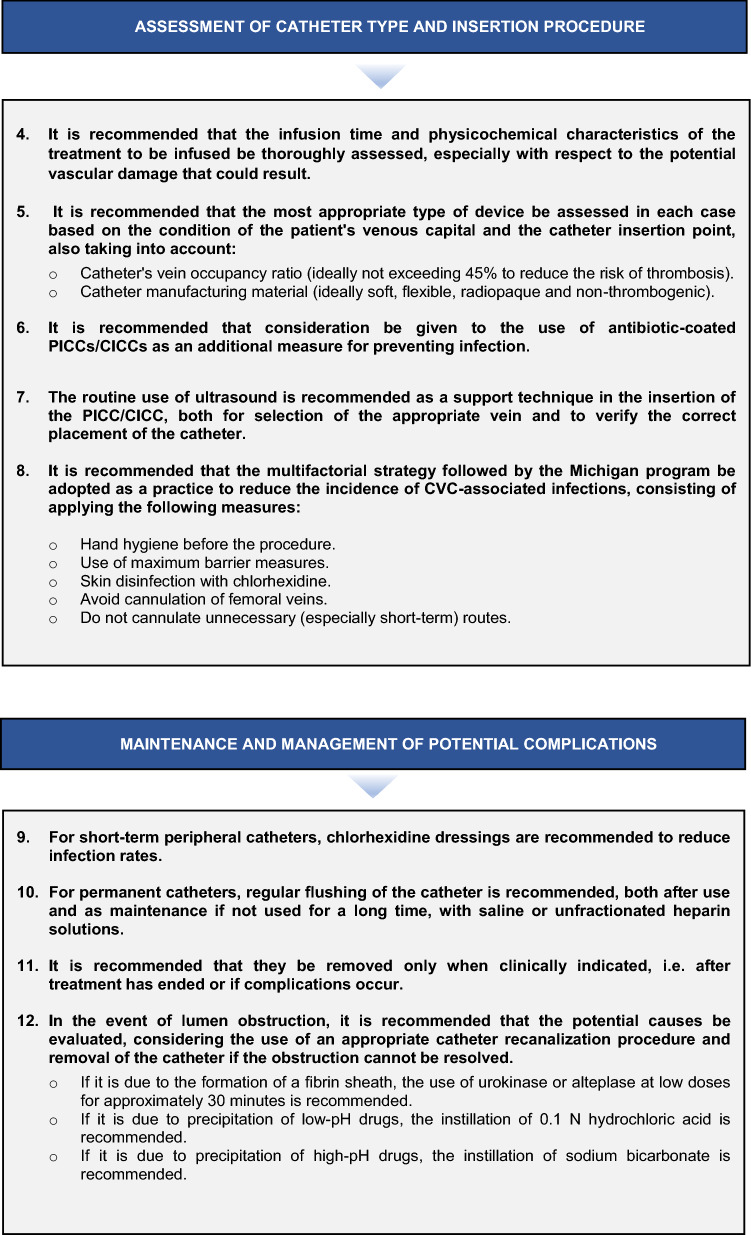
ECO-SEOM-SEEO recommendations for safe use of venous accesses in cancer patients

## Data Availability

The authors declare that all data and materials, as well as the application of the software used, support their published claims and meet the required standards.

## Appendix

See Tables 2, 3, and 4.

**Table 2:** VIA scale of assessment of the condition of the patient's venous capital using objective scales [43]

VIA grade	Possible puncture points	Catheter gauge (at least)	Risk of extravasation	Ease of venipuncture	Infusion status
Grade 1	6	18G	Remote	Very easy	Flows fast and without resistance
Grade 2	4	20G	Low	Easy	Offers resistance
Grade 3	3	22G	Possible	Not easy	Tendency to resistance in prolonged infusion
Grade 4	1	24G	High	Difficult	High risk of phlebitis
Grade 5	0	No possibilities	Very high	Very difficult	Very high risk of phlebitis

^*^The Venous International Assessment (VIA) scale establishes 5 grades of venous condition assessment based on possible puncture points, catheter gauge, risk of extravasation, ease of performing the technique and intravenous medication status

**Table 3:** Advantages and disadvantages of the main CVCs used in cancer patients

Type of CVC	Advantages	Disadvantages
PICC	Possibility of ultrasound-guided insertion to reduce complications Easy removal of the catheter after use or due to complications Implantable by bedside Nursing staff Low-gauge catheters available to minimize thrombosis rates Low incidence of catheter-related infections	High risk of thrombosis from displacement and/or incorrect positioning of the tip Higher risk of deep vein thrombosis than with CICC
Reservoir	Low incidence of infections and catheter obstruction Comfort and better perception of the image by the patient	Requires surgical procedure for insertion and removal High cost associated with the resources required for its implementation
Tunneled CICC	Possibility of ultrasound-guided insertion to reduce complications	Requires surgical procedure for insertion and removal Higher risk of infections than with PICC Higher risk of central vein thrombosis that with PICC

**Table 4:** Classification of the main cytotoxic agents [23, 44]

Vesicant	Irritant	Non-vesicant*
DNA-binding Amsacrine Carmustine Dacarbazine Dactinomycin Daunorubicin Doxorubicin Epirubicin Idarubicin Mechlorethamine Nitrogen mustard Mitomycin Streptozotocin Treosulfan	Bendamustine Carboplatin Cisplatin Etoposide Fluorouracil Ifosfamide Irinotecan Liposomal daunorubicin Liposomal doxorubicin Melphalan Methotrexate Mitoxantrone Oxaliplatin Temsirolimus Teniposide Topotecan Trastuzumab emtansine Aflibercept	Arsenic trioxide Asparaginase Bleomycin Bortezomib Cladribine Cyclophosphamide Cytarabine Eribulin Fludarabine Gemcitabine Monoclonal antibodies Paclitaxel albumin Pemetrexed Pentostatin Raltitrexed Thiotepa
Non-DNA-binding Cabazitaxel Docetaxel Paclitaxel Trabectedin Vinblastine Vincristine Vindesine Vinflunine Vinorelbine		
